# Effects of Roundup and its main component, glyphosate, upon mammalian sperm function and survival

**DOI:** 10.1038/s41598-020-67538-w

**Published:** 2020-07-03

**Authors:** Chiara Nerozzi, Sandra Recuero, Giovanna Galeati, Diego Bucci, Marcella Spinaci, Marc Yeste

**Affiliations:** 1 Biotechnology of Animal and Human Reproduction (TechnoSperm), Institute of Food and Agricultural Technology, University of Girona, Girona, Spain; 2 Unit of Cell Biology, Department of Biology, Faculty of Sciences, University of Girona, C/Maria Aurèlia Campany, 69, Campus Montilivi, 17003 Girona, Spain; 3 Department of Veterinary Medical Sciences (DIMEVET), University of Bologna, Bologna, Italy

**Keywords:** Cell biology, Animal biotechnology, Reproductive biology, Environmental impact

## Abstract

The wide use of glyphosate-based herbicides (GBHs) has become a matter of concern due to its potential harmful effects on human health, including men fertility. This study sought to investigate, using the pig as a model, the impact of pure glyphosate and its most known commercial formulation, Roundup, on sperm function and survival. With this purpose, fresh commercial semen doses were incubated with different concentrations (0–360 µg/mL) of glyphosate (GLY; exp. 1) or Roundup, at the equivalent GLY concentration (exp. 2), at 38 °C for 3 h. Glyphosate at 360 µg/mL significantly (*P* < 0.05) decreased sperm motility, viability, mitochondrial activity and acrosome integrity but had no detrimental effect at lower doses. On the other hand, Roundup did significantly (*P* < 0.05) reduce sperm motility at ≥ 5 µg/mL GLY-equivalent concentration; mitochondrial activity at ≥ 25 µg/mL GLY-equivalent concentration; and sperm viability and acrosome integrity at ≥ 100 µg/mL GLY-equivalent concentration as early as 1 h of incubation. In a similar fashion, GLY and Roundup did not inflict any detrimental effect on sperm DNA integrity. Taken together, these data indicate that, while both glyphosate and Roundup exert a negative impact on male gametes, Roundup is more toxic than its main component, glyphosate.

## Introduction

Glyphosate (GLY) is the active ingredient of all glyphosate-based herbicides (GBHs), including the most famous commercial formulation, Roundup (R). Despite GBHs being currently used worldwide, not only does this massive usage represent a risk for farmers but also for the general population, as environmental contamination with glyphosate affects water and food consumption^1,2^. For this reason, the use of GBHs has become a matter of concern for public health, and much debate has been raised about their potential carcinogenicity and negative impact on neurologic, gastroenteric, endocrine and reproductive systems^3–9^.

Previous research has confirmed that, at low doses, glyphosate acts as an endocrine disruptor in mammals altering hormonal function^10,11^. In particular, it has been suggested that GBHs can interfere with steroidogenesis in different ways, such as downregulating the expression of the steroidogenic acute regulatory protein (STAR), disrupting cytochrome P450 aromatase^10–12^, impairing the expression of oestrogen-regulated genes, or interacting with both androgen and oestrogen receptors^6,10–11,13^. Furthermore, GBHs have been purported to induce redox imbalance, causing Ca^2+^ overload and depletion of the antioxidant defence systems^14,15^. Since they often contain traces of heavy metals that can reach up to 80 ppb, such as arsenic, chromium, cobalt, lead and nickel, GBHs are also known to be cytotoxic and act as endocrine disruptors^16^.

Concerning the effects on male fertility, Anifandis et al*.* found that addition of 0.36 µg/mL glyphosate causes a significant reduction in progressive motility of human spermatozoa after 1 h incubation^17^. Furthermore, Clair et al*.* reported that glyphosate at 1,800 µg/mL is cytotoxic for testicular germ cells and, to a lesser extent, for Leydig cells^18^. It is worth mentioning that the aforementioned concentration is half of the one utilised to dilute the herbicide. Remarkably, signs of endocrine disruption, such as a decrease of 35% in testosterone serum levels, are detected at much lower glyphosate concentration (0.36 µg/mL)^18^. Using the zebrafish as a model, Lopes et al*.* reported that 24 h after adding feeding with glyphosate, there was a reduction in sperm motility (5 mg/mL) and in mitochondrial activity and DNA integrity (10 mg/mL)^19^.

Most of the research conducted in the last years has been focused on commercial glyphosate formulations, since they seem to exert more detrimental side-effects than glyphosate alone, possibly because formulants are not inert compounds at all^10,13,20–27^. As far as Roundup is concerned, it has been demonstrated that very low concentrations of this commercial product alter steroidogenesis^12^, and that it induces a notable cytotoxic effect on all testicular rat cells after incubation at 0.1% (corresponding to 360 µg/mL glyphosate) for 24 h^18^. This concentration is ten times lower than that recommended for agricultural use. According to the only in vitro experiment conducted thus far, incubation of human sperm with 1 µL/mL Roundup (corresponding to a glyphosate concentration of 0.36 µg/mL) for 1 h causes a drop in progressive motility and a depletion in mitochondrial activity^17^. Moreover, in vivo studies conducted in a fish species (*Jenynsia multidentata*) showed that exposure to Roundup, at a corresponding glyphosate concentration of 0.5 µg/mL, for 24 h decreases sperm motility and concentration^28^. In rats, exposure to 5 µg/mL Roundup for 8 days increases the proportions of morphologically abnormal spermatozoa and alters nuclear integrity^29^. In addition, when rats are exposed to Roundup during the prepubertal period, there is a decrease in serum testosterone levels and changes in testicular morphology occur^30,31^. Perinatal exposure leads to an increase in serum concentrations of testosterone, oestradiol and luteinizing hormone, inducing an early onset of puberty and sexual behavioural changes in the male offspring^32^. When the time of exposure lasts from the perinatal period to lactation, male offspring rats suffer from a drop in daily sperm production and show morphological abnormalities^33^. Finally, Roundup administered from the prenatal period to adulthood at an acceptable daily intake (ADI) dose of 1.75 mg/kg of body weight per day is enough to induce an alteration in rat reproductive developmental parameters, such as the increase of anogenital distance, which is a marker of prenatal endocrine disruption^34^.

Against this background, the aim of this work was to investigate, for the first time in the same study, the effects of different concentrations of glyphosate and Roundup on mammalian sperm following exposure for 1 h and 3 h at 38°. We used the pig as a model, and tested concentrations ranging from either 360 µg/mL GLY or 0.1% Roundup (equivalent to 360 µg/mL GLY), which seem to be cytotoxic according to Clair et al*.*^18^, to 70-fold lower. All Roundup concentrations are expressed as GLY-equivalent concentration.

## Results

### Experiment 1: Effects of glyphosate on sperm quality and functional parameters

**Figure 1:**
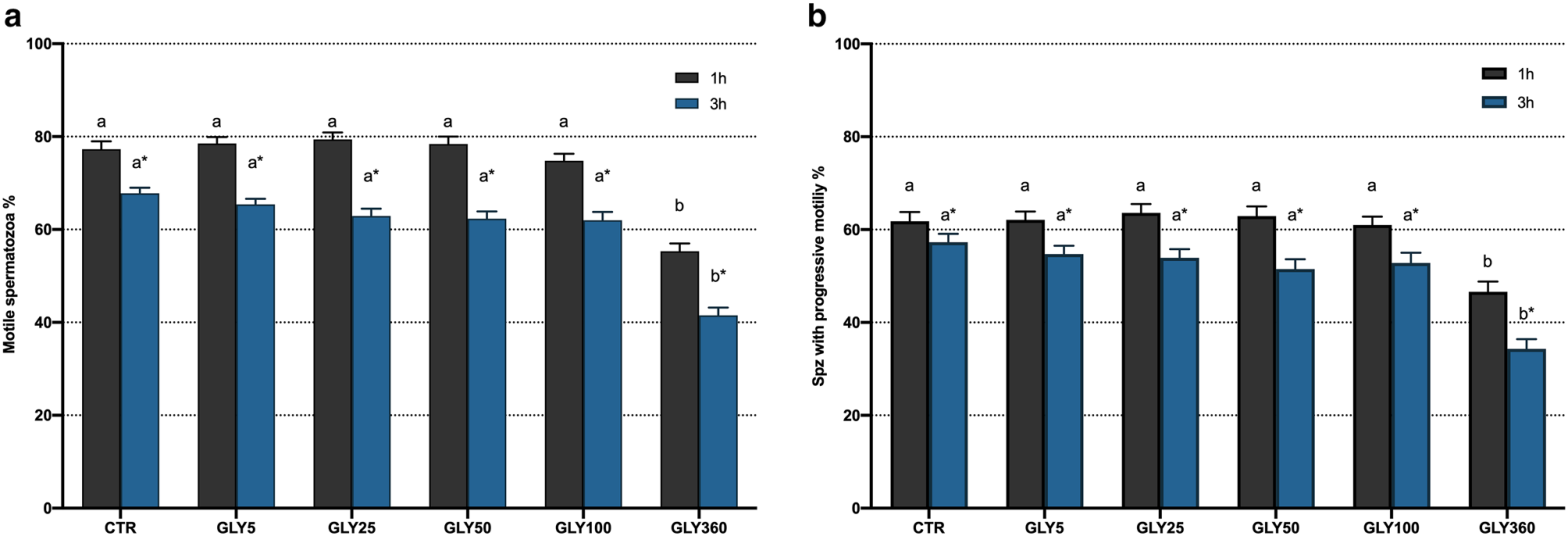
Effects of exposure to 0, 5, 25, 50, 100 and 360 µg/mL glyphosate on total (**a**) and progressive (**b**) sperm motility evaluated through CASA system. Different letters (*a*,* b*) represent significant (*P* < 0.05) differences between treatments. (*) represents significant (*P* < 0.05) differences between incubation times within a given treatment. CTR: control, sperm sample without addition of glyphosate; Spz: spermatozoa. Data are shown mean ± SEM.

**Figure 2:**
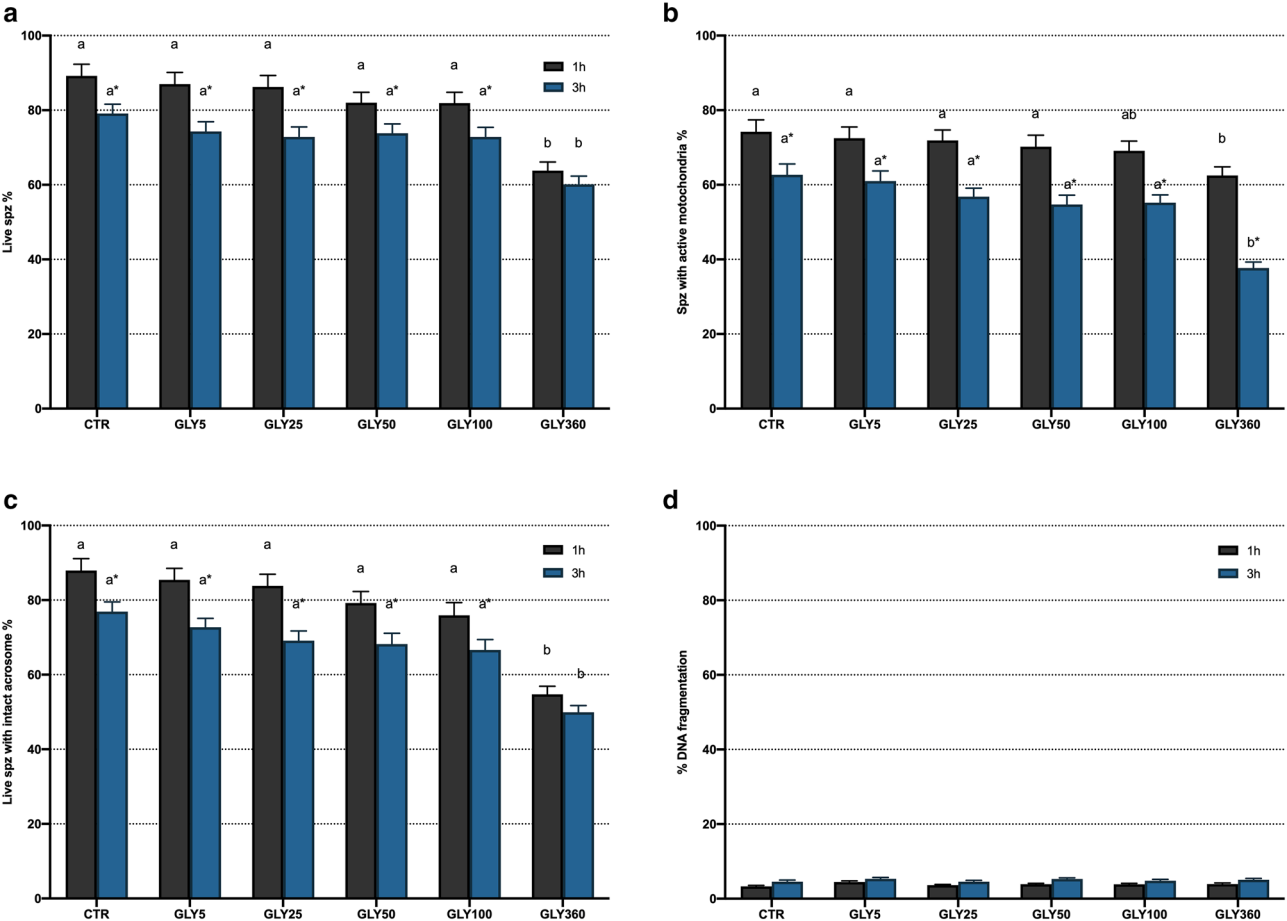
Effects of exposure to 0, 5, 25, 50, 100 and 360 µg/mL glyphosate on viability (**a**), percentage of spermatozoa with high mitochondrial membrane potential (**b**), acrosome integrity (**c**) and DNA fragmentation (**d**). Different letters (*a*,* b*) represent significant (*P* < 0.05) differences between treatments. (*) represents significant (*P* < 0.05) differences between incubation times within a given treatment. CTR: control, sperm sample without addition of glyphosate; Spz: spermatozoa. Data are shown mean ± SEM.

Compared to the control, addition of 360 µg/mL glyphosate significantly (*P* < 0.05) decreased total and progressive motility, viability, mitochondrial activity and acrosome integrity after 1 h and 3 h of incubation at 38 °C. In contrast, no significant differences between the control and lower glyphosate concentrations were observed. On the other hand, DNA fragmentation analysis showed no differences (*P* > 0.05) between the control and treatments after either 1 h or 3 h of incubation at 38 °C (Figs. 1, 2).

### Experiment 2: Effects of Roundup on sperm parameters

**Figure 3:**
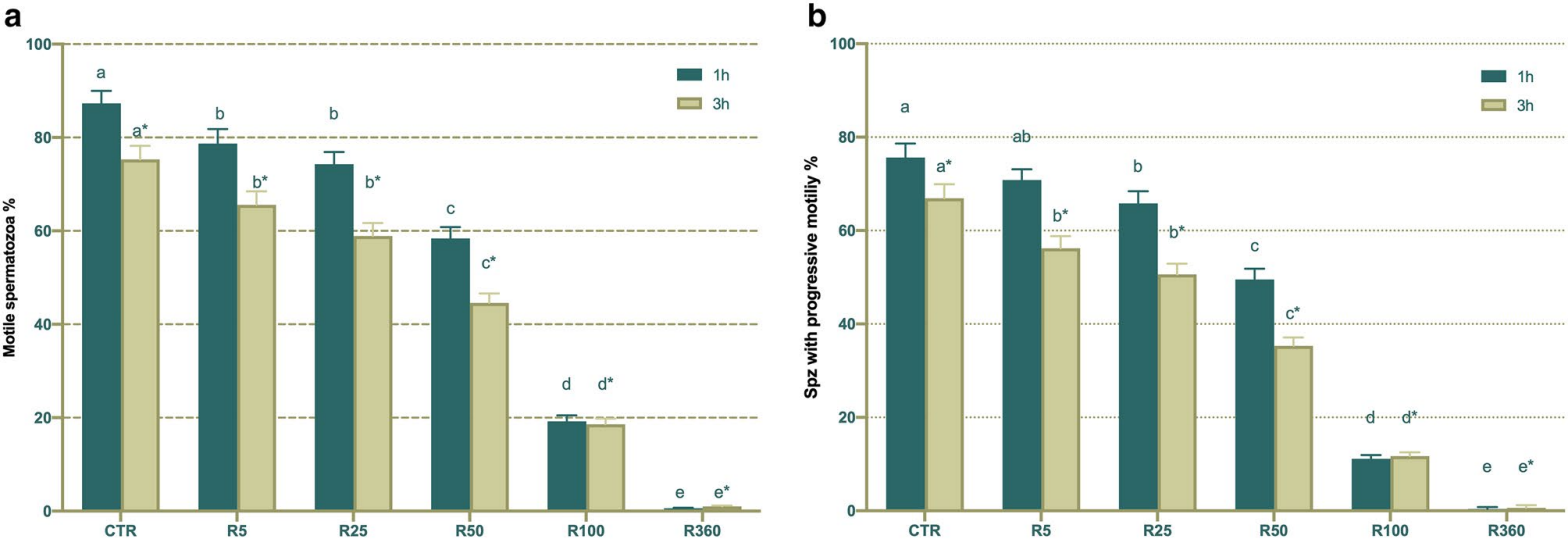
Effects of exposure to Roundup (at concentrations equivalent to glyphosate: 0, 5, 25, 50, 100 and 360 µg/mL) on total (**a**) and progressive (**b**) motility acquired by CASA system. Different letters (*a, b*) represent significant (*P* < 0.05) differences between treatments. (*) represents significant (*P* < 0.05) differences between incubation times within a given treatment. CTR: control, sperm sample without addition of Roundup; Spz: spermatozoa. Data are shown mean ± SEM.

As shown in Fig. 3, total sperm motility was significantly (*P* < 0.05) reduced in all Roundup concentrations (≥ 5 µg/mL GLY-equivalent) after 1 h and 3 h of incubation at 38 °C. In the case of progressive sperm motility, exposure to Roundup at concentrations ≥ 25 µg/mL GLY-equivalent significantly (*P* < 0.05) decreased this parameter after 1 h of incubation at 38 °C. In addition, concentrations ≥ 5 µg/mL GLY-equivalent showed significantly (*P* < 0.05) lower progressive sperm motility than the control after 3 h of incubation at 38 °C. Therefore, there was a dose-dependent impact of Roundup upon total and progressive sperm motility (Fig. 3). In addition, not only did Roundup have detrimental effects on total and progressive sperm motility, but also on kinematic parameters, as shown in Supplementary Table 2.

**Figure 4:**
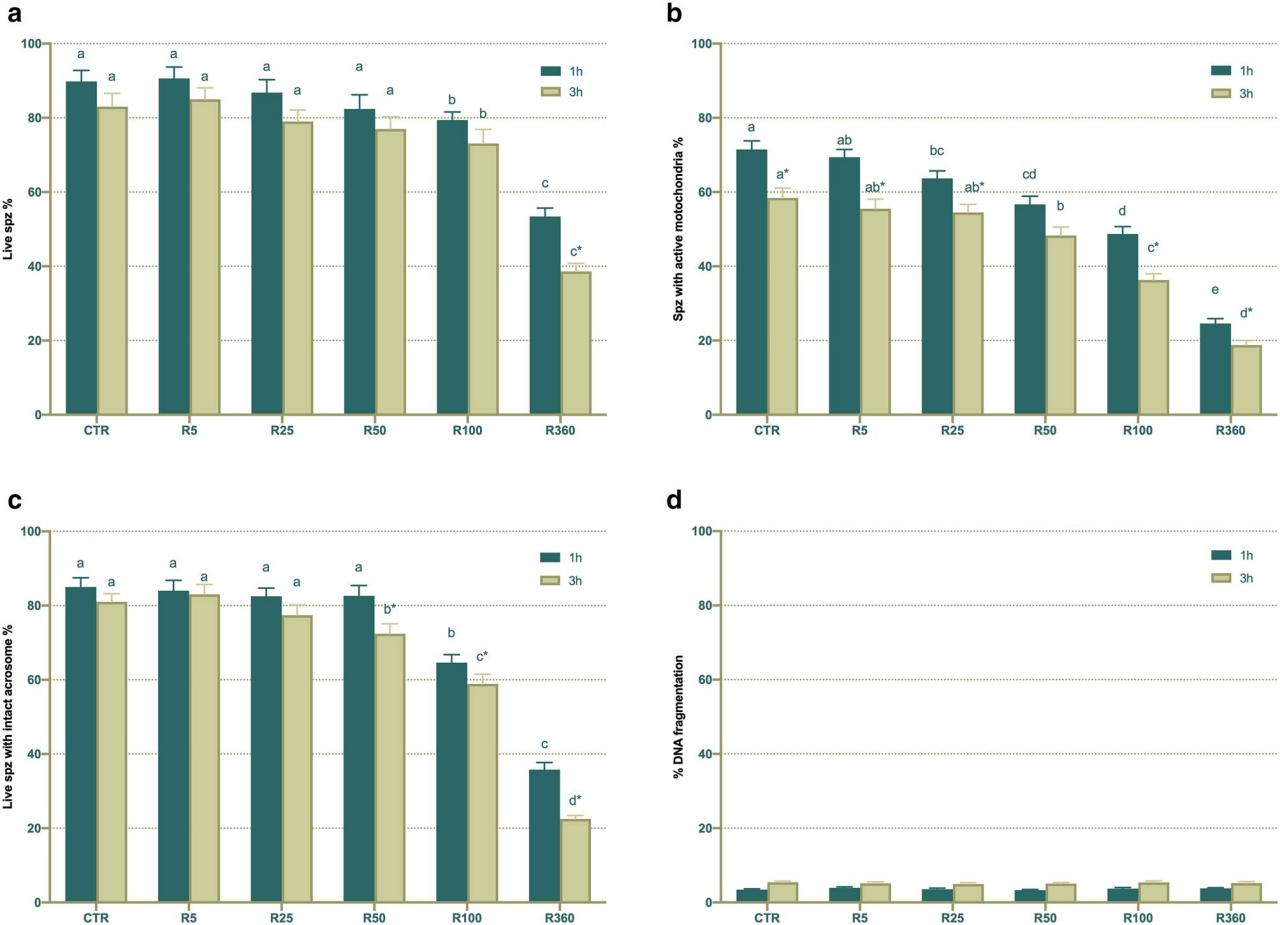
Effects of exposure to Roundup (at concentrations equivalent to glyphosate: 0, 5, 25, 50, 100 and 360 µg/mL) on sperm viability (**a**), percentage of spermatozoa with high mitochondrial membrane potential (**b**), acrosome integrity (**c**) and DNA fragmentation (**d**). Different letters represent significant (*P* < 0.05) differences between treatments. (*) represents significant (*P* < 0.05) differences between incubation times within a given treatment. CTR: control, sperm sample without addition of Roundup; Spz: spermatozoa. Data are shown mean ± SEM.

On the other hand, percentages of viable spermatozoa (SYBR14^+^/PI^-^) significantly (*P* < 0.05) decreased when semen samples were exposed to Roundup at concentrations equal or higher than 100 µg/mL GLY-equivalent. This reduction, which was observed after 1 h of incubation at 38 °C, persisted at 3 h (Fig. 4). As expected, exposure to Roundup also led to a significant (*P* < 0.05) decrease in the percentages of acrosome-intact spermatozoa after 1 h (≥ 100 µg/mL GLY-equivalent) and 3 h of incubation at 38 °C (≥ 50 µg/mL GLY-equivalent; Fig. 4).

Addition of semen samples with Roundup ≥ 25 µg/mL GLY-equivalent significantly (*P* < 0.05) reduced the percentage of spermatozoa with high MMP after 1 h of incubation at 38 °C. Furthermore, exposure to Roundup ≥ 50 µg/mL GLY-equivalent significantly (*P* < 0.05) decreased the percentage of spermatozoa with high MMP after 3 h of incubation at 38 °C (Fig. 4). In a similar fashion to that observed for sperm motility, viability, acrosome integrity and mitochondrial activity decreased in a dose-dependent manner. Finally, Roundup, like glyphosate, had no effect on sperm DNA integrity.

## Discussion

At present, there is a consistent body of literature suggesting that GBHs have negative effects on human health. However, it remains to be elucidated whether it is the main component of these products, i.e. glyphosate, or other compounds present in the commercial formulation of GBHs, which are the most toxic for the organism. In addition, although the detrimental effects of GBHs and glyphosate have been studied in animal models, with special reference to endocrine disruption, less attention has been paid on the effects of these products on mammalian sperm quality. For this reason, this study was the first to evaluate the effects of exposing mammalian spermatozoa to pure glyphosate or one of its most commercial formulations (Roundup) at different concentrations (0, 5, 25, 50, 100 and 360 µg/mL glyphosate, or glyphosate equivalent doses in the case of Roundup). We utilised the pig as a model, since not only is this animal species important for agriculture, but also for its use in biomedical research, due to its anatomical and physiological similarities with the human^35,36^. Moreover, and according to the 3Rs principle, sperm from mammalian species other than rodents have been demonstrated to have the potential to serve as a useful in vitro screening test for reproductive toxicology^37^.

Sperm motility, sperm viability, acrosome integrity, mitochondrial membrane potential, and DNA integrity were evaluated after 1 h and 3 h of incubation at 38 °C. On the one hand, while we observed that pure glyphosate decreased sperm motility (both total and progressive), viability and mitochondrial membrane potential, this only occurred at the highest concentration (360 µg/mL) and after both 1 h and 3 h of incubation. In contrast, no effects were observed at lower concentrations. Whilst the detrimental impact of exposing mammalian spermatozoa to glyphosate has also been reported in humans, our results differ from those obtained by Anifandis et al*.*, as they found that progressive sperm motility decreased at a much lower concentration (0.36 µg/mL) after 1 h of incubation^17^. This discrepancy could be due to a species-specific sensitivity to glyphosate exposure and/or could be ascribed to the different procedures for sperm motility assessment; indeed, while we evaluated sperm motility through a CASA system, Anifandis et al*.*^17^ assessed human semen motility according to WHO 2010 guidelines under a phase-contrast microscope. With regard to DNA fragmentation, we found that glyphosate samples did not differ from the control, which in this case did agree with Anifandis et al*.*^17^.

The negative impact on quality and functional parameters was much more apparent when spermatozoa were exposed to Roundup. This finding confirmed previous research on human JEG3 and embryonic kidney 293 cells indicating that commercial formulations are far more deleterious than glyphosate itself^16,38^. Moreover, we observed that Roundup was detrimental for sperm in a concentration-dependent manner, as total sperm motility was impaired at the lowest concentration (5 µg/mL) and progressive sperm motility was significantly reduced at ≥ 25 µg/mL. These results agree with those of Anifandis et al*.*^39^, who found that progressive sperm motility in humans is negatively influenced by the presence of 1 µg/mL Roundup (corresponding to a glyphosate concentration of 0.36 µg/mL). Herein, however, we found that detrimental effects occur at higher doses in pig sperm, possibly due, again, to a species-dependent sensitivity to Roundup exposure and/or to the different methods used to assess sperm motility^17^. Moreover, and in agreement with Anifandis et al*.*^39^, the decrease in mitochondrial membrane potential was concomitant with that of total sperm motility, as incubation with 25 µg/mL Roundup for 1 h reduced mitochondrial activity. The detrimental impact of GBHs on mitochondrial activity has already been demonstrated in *Caenorhabditis elegans*, as exposing these animals to 3% TouchDown (expressed as % glyphosate; TouchDown Hitech, formulation of 52.3% glyphosate) leads to a decrease in mitochondrial respiration, oxygen consumption and ATP production, and an increase in intracellular levels of hydrogen peroxide^40^. These toxic effects have been suggested to be exerted through the impairment of the electron transport chain function via inhibition of Complex II (succinate dehydrogenase)^40,41^. Moreover, Roundup has been demonstrated to reduce the activity of mitochondrial succinate dehydrogenase in an immature mouse Sertoli TM4 cell line at a concentration as low as 0.001%, with heavier toxic effect than glyphosate alone^42^. By studying rat liver mitochondria, Peixoto et al*.* demonstrated the capacity of GBHS to alter mitochondrial bioenergetics at concentrations ≥ 5 mM glyphosate^43^. Our results are in agreement with the aforementioned studies and confirm that the compounds present in commercial herbicides may potentiate mitochondrial perturbation. However, it is worth mentioning that, in this study, spermatozoa considered to exhibit high mitochondrial membrane potential included heterogeneous sperm populations. These sperm populations went from cells with very high mitochondrial membrane potential, which showed orange fluorescence when stained with JC1, and with high or intermediate membrane potential, in which JC1 emitted in both orange and green along the mid-piece. Therefore, further studies should also evaluate the impact of glyphosate and Roundup on the separate sperm populations that show different green and orange fluorescence intensities following JC1-staining.

With regard to sperm viability, 100 µg/mL Roundup significantly reduced the percentages of SYBR14^+^/PI^-^ spermatozoa after 1 h of incubation at 38 °C. These results were in accordance with those obtained following evaluation of acrosome integrity, as percentages of spermatozoa exhibiting a non-intact acrosome were higher after 1 h and 3 h of exposure to 100 and 50 µg/mL Roundup. Previous studies confirmed the negative impact of Roundup on cell viability. Richard et al*.* reported its toxicity for human placental JEG3 cells at a concentration of 0.4% after 1 h of incubation^10^, and Clair et al*.* found signs of Roundup toxicity in both Leydig and Sertoli rat cells with a 0.1% concentration after 24 h^18^. The same result obtained by Clair et al*.* was confirmed by Vanlaeys et al*.* in an immature mouse Sertoli TM4 cell line TM4^42^.

It is worth noting that all sperm quality and functional parameters affected by the exposure to Roundup showed a clear dose-dependent trend. Moreover, as observed by Anifandis et al*.* in human sperm^39^, most of the induced damage was already apparent after 1 h of incubation at 38 °C, suggesting that the negative effects induced by Roundup could occur rapidly during the first hour of exposure. This quick action of Roundup, which contrasts with that of glyphosate alone, was already observed following 90-min exposure of human embryonic kidney cells (HEK 293) to different glyphosate-based herbicides, including two different Roundup formulations (Roundup WeatherMAX and Roundup Classic) that are very similar to Roundup Bioflow^16^.

To the best of our knowledge, this study represents the first evaluation and comparison of biological activities of glyphosate and Roundup on mammalian spermatozoa. Overall, our results suggest that, while both glyphosate and Roundup induce toxic effects on mammalian sperm function and survival, Roundup has much more detrimental impact than glyphosate, even at equivalent concentrations of glyphosate. Therefore, the fact that Roundup is more toxic than pure glyphosate itself, causing more severe alterations than this active principle, confirms the hypothesis that formulants present in commercial products either boost glyphosate toxicity or are harmful themselves^10,13,20–27^. Based on these results, not only should the perniciousness of glyphosate be evaluated when handling glyphosate-based herbicides, but also that of the other compounds. Therefore, whether bioaccumulation of these formulants, which are petroleum-derivatives, may have serious implications and cause chronic toxicity needs to be investigated. This is crucial given the growing concerns on the impact and safety of glyphosate and glyphosate-based herbicides.

On the other hand, one should bear in mind that, while this was a toxicological study testing high glyphosate and Roundup doses, environmental concentrations and levels in serum and urine recorded in recent publications are far lower than those tested herein^44,45^. Moreover, the biotransformation process that the compound undergoes inside the organism should also be taken into consideration, since glyphosate can be partially degraded prior to reaching male germ cells, which would make it less cytotoxic. In spite of all the aforementioned, it is clear from this study that the large use of glyphosate formulations, especially Roundup, may entail a risk for male fertility; hence, further research aimed at clarifying the effects and toxicity of each compound is much warranted.

Based on our results, it can be hypothesized that the toxic effect of these pesticides may be linked to an impairment in mitochondrial activity and a subsequent decrease in ATP production and/or alterations in the redox balance, which impact cell motility and plasma membrane stability. In spite of this, DNA integrity seem not to be altered either by Roundup or pure glyphosate. At present, the mechanism of action of GBHs remains unclear and needs to be investigated further.

In conclusion, the consequences of the massive use of glyphosate remains a matter concern for human health and food quality. Through using the mammalian spermatozoon, we found that both glyphosate and Roundup detrimentally affect cell function and survival, the latter being much more toxic than the former. This indicates that GBHs components other than glyphosate damage spermatozoa and may have a detrimental effect on fertilizing ability. Therefore, and in order to address the concerns on the use of GBHs properly, we suggest that all components present in the commercial formulation of glyphosate should also be tested individually. In addition, further research should address how each of these GBHs components damages the sperm cell.

## Methods

### Reagents

Unless otherwise specified, all chemicals were purchased from Sigma-Aldrich (Saint-Louis, MO, USA). The commercial formulation of glyphosate, Roundup Bioflow (containing 0.36 g/mL of glyphosate acid in the form of isopropylamine salts of glyphosate, 41.5%; water, 42.5%; and surfactant, 16%), was purchased from Monsanto Europe N.V. (Anversa, Belgium).

All fluorochromes were purchased from Invitrogen Molecular Probes (Thermo Fisher Scientific, Waltham, MA, USA) and diluted with dimethyl sulfoxide (DMSO).

### Seminal samples

A total of 10 different ejaculates coming from 10 separate Pietrain boars were used. Animals, aged between two and three years old, were collected by the hand-gloved method. Boar studs were healthy, stabled in climate-controlled buildings (Servicios Genéticos Porcinos, S.L., Roda de Ter, Spain), and fed an adjusted diet, with water being provided ad libitum. After collection, sperm-rich fractions were diluted to a final concentration of 30 × 10^6^ spermatozoa/mL with a commercial extender (Duragen, Magapor; Egea de los Caballeros, Zaragoza, Spain) and cooled to 17 °C. Ninety mL commercial doses were brought to our laboratory in about 45 min, inside a thermal container at 17 °C. It is worth mentioning that, since authors purchased seminal doses from the aforementioned local farm that operates under commercial, standard conditions, they did not manipulate any animal. Therefore, specific authorization from an Ethics Committee was not required to conduct this study.

### Experimental design

This work consisted of two separate experiments. In the first one, we investigated the impact of different glyphosate (GLY) concentrations (0, 5, 25, 50, 100 and 360 µg/mL) on sperm quality and functional parameters (30 × 10^6^ spermatozoa/mL). In the second experiment, pig semen was added with commercial glyphosate-based herbicide Roundup (R) at concentrations equivalent to the glyphosate ones tested in the first experiment. All Roundup treatments are expressed as glyphosate equivalent concentrations.

In each experiment, semen was added with glyphosate or Roundup at the aforementioned concentrations and then incubated at 38 °C for 3 h. After 1 h and 3 h of incubation, sperm motility, viability, mitochondrial membrane integrity, acrosome integrity and DNA fragmentation were evaluated.

### Evaluation of sperm motility

Sperm motility was evaluated with a commercial computer-assisted sperm analysis (CASA) system (Integrated Sperm Analysis System V1.0; Proiser, Valencia, Spain), following the settings described by Yeste et al*.*^46^. Briefly, samples were incubated at 38 °C for 5 min and a 5-µL drop was subsequently placed onto a pre-warmed Makler chamber. For every treatment, three replicates of 1,000 spermatozoa each were analysed. A sperm cell was considered to be motile when its average path velocity (VAP) was higher than 10 µm/s, and progressively motile when its straightness (STR) was higher than 45%.

### Analysis of sperm parameters with flow cytometry

Flow cytometry was used to determine sperm viability, acrosome integrity, mitochondrial activity and DNA fragmentation, and information in this section is given according to the recommendations of the International Society of Flow Cytometry^47^. In all assessments, sperm concentration was adjusted to 1 × 10^6^ spermatozoa/mL in a final volume of 0.5 mL, stained with fluorochromes and evaluated through a Cell Laboratory QuantaSC cytometer (Beckman Coulter, Fullerton, CA, USA). Sheath flow rate was set at 4.17 µL/min; electronic volume (EV) and side scatter (SS) were recorded in a linear mode (in EV vs. SS dot plots) for a minimum of 10,000 events per replicate. The analyser threshold was adjusted on the EV channel to exclude cell debris (particle diameter < 7 µm) and aggregates (particle diameter > 12 µm). Each parameter was evaluated twice for every treatment at both evaluation times. EV, SS, FL1, FL2 and FL3 were collected in List-mode Data files and cytometric histograms and dot plots were analysed with Lab QuantaSC MPL Software (version 1.0; Beckman Coulter).

### Evaluation of sperm viability

Sperm viability was assessed by using two fluorescent probes, SYBR14 and Propidium Iodide (PI), included in the LIVE/DEAD Sperm Viability Kit^48^. Following staining with 0.5 µL SYBR14 (final concentration: 100 nM) for 10 min at 38 °C in darkness, and then with 2.5 µL PI (final concentration: 12 μM) at the same conditions for 5 min, samples were analysed using two filters FL1 (SYBR14 detection) and FL3 (PI detection) and three different sperm populations were identified: (a) viable, green-stained spermatozoa (SYBR14^+^/PI^−^); (b) non-viable, red-stained spermatozoa (SYBR14^-^/PI^+^) and (c) non-viable spermatozoa that were stained both green and red (SYBR14^+^/PI^+^). Debris, non-stained particles appeared in the lower left quadrant (SYBR14^-^/PI^-^) and were subtracted from the total number of spermatozoa.

### Evaluation of acrosome integrity

Acrosome integrity was determined through staining with the lectin from *Arachis hypogaea* (PNA) conjugated with FITC (fluorescein isothiocyanate; PNA-FITC) and PI. Five hundred μL of each sperm sample was incubated with 0.5 μL PNA-FITC (final concentration: 1.25 mg/mL) and 2.5 μL PI (final concentration: 12 μM) for 10 min at 38 °C in darkness. Spermatozoa were then evaluated with the flow cytometer and two categories were distinguished: (a) viable spermatozoa with intact acrosome and plasma membrane (PNA-FITC^−^/PI^−^), and (b) spermatozoa that had damaged their plasma membrane and/or their acrosome, which included PNA-FITC^+^/PI^−^, PNA-FITC^+^/PI^+^, PNA-FITC^−^/PI^+^ populations. Debris, non-stained particles found in SYBR14/PI staining (i.e. SYBR14^−^/PI^−^) were subtracted from the PNA-FITC^-^/PI^-^ population and the other percentages were recalculated.

### Evaluation of mitochondrial membrane potential

Mitochondrial membrane potential (MMP) was evaluated with 5,5′,6,6′-tetrachloro-1,1′,3,3′tetraethylbenzimidazolylcarbocyanine iodide (JC1). Briefly, after incubating 500-μL aliquots with 0.5 μL JC-1 (final concentration: 3 μM) for 30 min at 38 °C in darkness, two sperm populations were distinguished: (a) spermatozoa with high MMP, which mainly emitted at the orange light spectrum as in these cells the stain aggregates and changes its fluorescence from green to orange; this population appeared in the upper half of FL1/FL2 dot-plots; and (b) spermatozoa with low MMP, which only emitted at the green light spectrum, as JC1 maintains its monomer status; this second population appeared in the lower half of FL1/FL2 dot-plots.

### Evaluation of DNA fragmentation

DNA fragmentation was assessed using the Sperm Chromatin Structure Assay (SCSA) test as modified by Morrell et al*.*^49^. Sperm samples were diluted to a final concentration of 2 × 10^6^ spermatozoa/mL with a buffer solution (TNE: 0.15 M NaCl, 0.01 M Tris–HCl, 1 mM EDTA, pH = 7.4). Two hundred µL of this solution were added with 400 µL of an acid-detergent solution (80 mM HCl, 150 mM NaCl, and 0.1% Triton X-100; pH = 1.2) on ice. After 30 s, samples were added with 1.2 mL acridine orange (AO) and incubated on ice for further 3 min. Afterwards, spermatozoa were evaluated with FL1 and FL3 filters for green and red fluorescence, respectively, and the following parameters were recorded: (a) percentage of DNA fragmentation (%DFI), which corresponded to the ratio between red (ssDNA) fluorescence and red (ssDNA) + green (dsDNA) fluorescence; (b) standard deviation of DFI; and (c) mean fluorescence intensity of ssDNA (Mean DFI).

### Statistical analyses

Statistical analyses were performed using a statistical package (IBM SPSS for Windows version 25.0; IBM Corp., Armonk, NY, USA). Data were first tested for normality and homogeneity of variances through Shapiro–Wilk and Levene tests, respectively. When required, data (x) were transformed using arcsine square root (arcsin √x) before a general mixed model, in which the between-subjects factor was the treatment (GLY or R concentrations) and the within-subjects factor was the incubation time at 38 °C (1 or 3 h), was run. Pair-wise comparisons were made with post-hoc Sidak test. When no transformation attained normal distribution and homoscedasticity, Scheirer–Ray–Hare and Mann–Whitney tests were used as non-parametric alternative models. In all cases, data are shown as mean ± standard error (SEM) and the minimal level of significance was set at *P* ≤ 0.05.

## Supplementary information

Supplementary file1 (DOCX 18 kb)

## Data Availability

Data are available from the authors on reasonable request.

## Abbreviations

CASA: Computer-assisted sperm analysis
JC1: 5,5′,6,6′-Tetrachloro-1,1′,3,3′tetraethylbenzimidazolylcarbocyanine iodide
GLY: Glyphosate
GBHs: Glyphosate-based herbicides
MMP: Mitochondrial membrane potential
PI: Propidium iodide
PNA: Lectin from *Arachis hypogaea*
3Rs: Replacement, reduction and refinement
SCSA: Sperm chromatin structure assay
