# Supplementary material for: Effects of Roundup and its main component, glyphosate, upon mammalian sperm function and survival
Source: Sci Rep. 2020 Jul 3;10:11026. doi: 10.1038/s41598-020-67538-w (PMC7335210; doi:10.1038/s41598-020-67538-w)
Supplement: Supplementary file 1 — Supplementary file1 (DOCX 18 kb) [file 41598_2020_67538_MOESM1_ESM.docx]

**Title**

Effects of Roundup and its main component, Glyphosate, upon mammalian sperm function and survival

**Authors**

Chiara Nerozzi^1, 2, 3^, Sandra Recuero^1, 2^, Giovanna Galeati^3^, Diego Bucci^3^, Marcella Spinaci^3^, Marc Yeste^1, 2, *^

**Supplementary Table 1.** Effects of exposure to 0, 5, 25, 50, 100 and 360 µg/mL glyphosate on kinematic sperm parameters (VCL, VAP, VSL, LIN, STR, WOB, ALH and BCF) recorded by CASA system. Results are expressed as mean ± SEM. Different letters represent significant (*P*≤0.05) differences between treatments. (*) indicates significant (*P*≤0.05) differences between incubation times (1 h *vs.* 3 h) within a given treatment.

| **1h** |  | CTR | Gly 5 μg/mL | Gly 25 μg/mL | Gly 50 μg/mL | Gly 100 μg/mL | Gly 360 μg/mL |
| --- | --- | --- | --- | --- | --- | --- | --- |
|  | **VCL** | 72.4 ± 2.9 ^a^ | 73.5 ± 1.9 ^a^ | 78.4 ± 2.2 ^a^ | 75.3 ± 1.7 ^a^ | 77.6 ± 1.6 ^a^ | 71.7 ± 0.9 ^a^ |
|  | **VSL** | 47.1 ± 2.2 ^a^ | 47.9 ± 1.1 ^a^ | 50.9 ± 0.9 ^a^ | 49.8 ± 0.7 ^a^ | 52.3 ± 0.6 ^a^ | 51.1 ± 1.0 ^a^ |
|  | **VAP** | 61.5 ± 2.7 ^a^ | 62.5 ± 1.7 ^a^ | 66.5 ± 1.7 ^a^ | 64.2 ± 1.4 ^a^ | 66.1 ± 1.3 ^a^ | 60.6 ± 0.9 ^a^ |
|  | **LIN** | 66.2 ± 0.9 ^a^ | 66.2 ± 0.8 ^a^ | 66.9 ± 1.2 ^a^ | 67.6 ± 1.0 ^a^ | 68.6 ± 1.0 ^a^ | 71.6 ± 1.0 ^a^ |
|  | **STR** | 77.9 ± 0.8 ^a^ | 77.7 ± 0.8 ^a^ | 78.0 ± 1.0 ^a^ | 78.8 ± 0.9 ^a^ | 80.0 ±0.8 ^a^ | 84.4 ± 0.7 ^a^ |
|  | **WOB** | 85.0 ± 0.2 ^a^ | 85.1 ± 0.3 ^a^ | 85.4 ± 0.4 ^a^ | 85.6 ± 0.3 ^a^ | 85.6 ± 0.3 ^a^ | 84.6 ± 0.6 ^a^ |
|  | **ALH** | 2.3 ± 0.3 ^a^ | 2.3 ± 0.3 ^a^ | 2.4 ± 0.3 ^a^ | 2.4 ± 0.3 ^a^ | 2.4 ± 0.2 ^a^ | 2.3 ± 0.2 ^a^ |
|  | **BCF** | 8.1 ± 0.2 ^a^ | 8.2 ± 0.1 ^a^ | 8.2 ± 0.1 ^a^ | 8.3 ± 0.1 ^a^ | 8.3 ± 0.1 ^a^ | 8.4 ± 0.2 ^a^ |
| **3h** |  | CTR | Gly 5 μg/mL | Gly 25 μg/mL | Gly 50 μg/mL | Gly 100 μg/mL | Gly 360 μg/mL |
|  | **VCL** | 73.2 ± 2.5 ^a^ | 77.4 ± 1.3 ^a^ | 71.4 ± 1.4 ^a^ | 75.6 ± 1.3 ^a^ | 77.5 ± 1.5 ^a^ | 69.2 ± 0.8 ^a^ |
|  | **VSL** | 52.7 ± 2.2 ^a^ | 55.0 ± 1.6 ^a^* | 51.0 ± 1.3 ^a^ | 53.0 ± 1.4 ^a^ | 56.1 ± 1.8 ^a^ | 49.4 ± 1.4 ^a^ |
|  | **VAP** | 63.9 ± 2.3 ^a^ | 66.6 ± 1.5 ^a^ | 61.4 ± 1.5 ^a^ | 64.3 ± 1.5 ^a^ | 66.8 ± 1.9 ^a^ | 57.2 ± 1.1 ^a^ |
|  | **LIN** | 74.3 ± 1.4 ^a^* | 70.5 ± 0.9 ^a^ | 71.4 ± 0.7 ^a^ | 71.4 ± 0.7 ^a^ | 71.8 ± 1.0 ^a^ | 71.0 ± 1.2 ^a^ |
|  | **STR** | 84.6 ± 1.2 ^a^* | 82.2 ± 0.6 ^a^* | 83.3 ± 0.6 ^a^* | 82.6 ± 0.7 ^a^ | 83.8 ± 0.7 ^a^ | 85.8 ± 0.7 ^a^ |
|  | **WOB** | 87.6 ± 0.4 ^a^* | 85.6 ± 0.4 ^ab^ | 85.7 ± 0.4 ^ab^ | 84.5 ± 0.5 ^ab^ | 85.5 ± 0.7 ^ab^ | 82.4 ± 0.7 ^b^ |
|  | **ALH** | 2.3 ± 0.2 ^a^ | 2.3 ± 0.2 ^a^ | 2.1 ± 0.1 ^a^* | 2.3 ± 0.2 ^a^ | 2.2 ± 0.1 ^a^ | 2.2 ± 0.2 ^a^ |
|  | **BCF** | 8.5 ± 0.2 ^a^* | 8.5 ± 0.2 ^a^* | 8.4 ± 0.2 ^a^ | 8.4 ± 0.2 ^a^ | 8.5 ± 0.2 ^a^ | 8.6 ± 0.3 ^a^ |

**Supplementary Table 2.** Effects of exposure to 0, 5, 25, 50, 100 and 360 µg/mL Roundup on kinematic sperm parameters (VCL, VAP, VSL, LIN, STR, WOB, ALH and BCF) recorded by CASA system. Results are expressed as mean ± SEM. Different letters represent significant (*P*≤0.05) differences between treatments. (*) indicates significant (*P*≤0.05) differences between incubation times (1 h *vs.* 3 h) within a given treatment.

| **1h** |  | CTR | R 5 μg/mL | R 25 μg/mL | R 50 μg/mL | R 100 μg/mL | R 360 μg/mL |
| --- | --- | --- | --- | --- | --- | --- | --- |
|  | **VCL** | 58.2 ± 1.8^a^ | 57.5 ± 1.7^a^ | 55.6 ± 1.9^a^ | 52.7 ± 1.7^a^ | 43.9 ± 2.0^b^ | 27.1 ± 1.0^c^ |
|  | **VSL** | 40.8 ± 2.5^a^ | 39.3 ± 2.6^ab^ | 35.1 ± 1.5^ab^ | 33.1 ± 1.7^b^ | 21.4 ± 1.5^c^ | 3.9 ± 0.7^d^ |
|  | **VAP** | 49.2 ± 2.7^a^ | 46.9 ± 2.2^ab^ | 43.6 ± 3.0^ab^ | 40.9 ± 2.4^b^ | 30.5 ± 2.0^c^ | 11.1 ± 1.0^d^ |
|  | **LIN** | 70.3 ± 1.8^a^ | 71.7 ± 1.6^a^ | 65.9 ± 2.2^a^ | 65.4 ± 2.6^a^ | 44.5 ± 2.8^b^ | 17.2 ± 1.4^c^ |
|  | **STR** | 82.7 ± 1.1^a^ | 84.4 ± 1.0^a^ | 82.9 ± 1.5^a^ | 80.6 ± 1.8^a^ | 62.2 ± 1.8^b^ | 38.4 ± 2.6^c^ |
|  | **WOB** | 84.8 ± 1.3^a^ | 84.8 ± 1.1^a^ | 78.9 ± 2.8^a^ | 80.2 ± 2.4^a^ | 65.1± 2.5^b^ | 43.9 ± 1.6^c^ |
|  | **ALH** | 2.0 ± 0.1^a^ | 3.0 ± 0.1^a^ | 1.9 ±0.1^a^ | 1.7 ± 0.1^b^ | 1.7 ± 0.2^b^ | 1.1 ± 0.5^c^ |
|  | **BCF** | 8.1 ± 0.1^a^ | 8.1 ± 0.3^a^ | 7.0 ± 0.2^a^ | 7.4 ± 0.2^a^ | 4.3 ± 0.2^b^ | 0.8 ± 0.1^c^ |
| **3h** |  | CTR | R 5 μg/mL | R 25 μg/mL | R 50 μg/mL | R 100 μg/mL | R 360 μg/mL |
|  | **VCL** | 57.6 ± 2.6^a^ | 54.6 ± 2.2^ab^ | 52.6 ± 1.8^ab^ | 46.2 ± 1.8^b^ | 46.2 ± 1.4^b^ | 23.1 ± 1.3^c*^ |
|  | **VSL** | 38.5 ± 1.7^a^ | 38.4 ± 2.2^a^ | 34.1 ± 2.3^a^ | 31.4 ± 1.2^b^ | 18.7 ± 1.2^c*^ | 3.6 ± 0.7^d^ |
|  | **VAP** | 47.9 ± 1.8^a^ | 46.7 ± 2.1^a^ | 41.9 ± 2.5^ab^ | 39.6 ± 1.9^b^ | 27.0 ± 1.9^c*^ | 9.9 ± 0.5^d*^ |
|  | **LIN** | 66.1 ± 1.9^a^ | 66.3 ± 2.8^a^ | 59.7 ± 2.9^a^ | 63.8 ± 2.4^a^ | 37.9 ± 1.8^b^ | 12.4 ± 1.0^c^ |
|  | **STR** | 80.2 ± 1.3^a^ | 81.5 ± 1.5^a^ | 74.8 ± 2.0^a^ | 79.7 ± 1.5^a^ | 58.8 ± 2.2^b^ | 28.6 ± 2.2^c^ |
|  | **WOB** | 82.2 ± 1.1 ^a^ | 81.0 ± 2.2^a^ | 77.3 ± 2.6^a^ | 78.2 ± 2.2^b^ | 56.9 ± 2.6^b^ | 41.7 ± 1.4^c^ |
|  | **ALH** | 1.9 ± 0.0^a^ | 1.8 ± 0.1^a^ | 2.0 ± 0.1^a^ | 1.8 ± 0.1^b^ | 1.6 ± 0.1^b^ | 0.7 ± 0.1^c^ |
|  | **BCF** | 8.0 ± 0.1^a^ | 8.0 ± 0.1^a^ | 7.8 ± 0.2^a^ | 7.2 ± 0.3^a^ | 4.8 ± 0.1^b^ | 0.5 ± 0.1^c^ |
